# Supplementary material for: Patterns of Intron Gain and Loss in Fungi
Source: PLoS Biol. 2004 Nov 30;2(12):e422. doi: 10.1371/journal.pbio.0020422 (PMC532390; doi:10.1371/journal.pbio.0020422)
Supplement: Table S1 — Also available at http://genes.mit.edu/NielsenEtAl/. (4.3 MB ZIP). [file pbio.0020422.st001.zip › NielsenEtAl/html/1314.html]

AN5790.1.NCU00775.1.MG01995.1.FG05733.1


```
 CLUSTAL W (1.82) Multiple Sequence Alignments - Introns Inserted


Sequence 1: NCU00775.1	400 aa
Sequence 2: FG05733.1	378 aa
Sequence 3: AN5790.1	386 aa
Sequence 4: MG01995.1	381 aa
Alignment Length: 407 aa
Number Identitical Residues: 273 aa
Alignment Score (without introns) 11351


MG01995.1 	MLGTSRSAQVS~----------S~G~IN------SLIQID0-ASRT~FA--------TVQ
NCU00775.1	MLSRNSMVCLL~ILLLLLLSWLL0R~LARAVAQ----PTP~RLARG~FASVQDTPAQTLQ
FG05733.1 	MLSRGSLRS--~----------A~Q0LLRGVAK----QQP~QLARS~FA--------TVQ
AN5790.1  	MFGTRTVQPAK0----------S~L~FRRAAAQPSIVRSP~IAARS1FA--------TVQ
          	*:.                       :  . :..:        :*  **        *:*

MG01995.1 	SDIFKPSKYGGKYTVTLIPG~DGIGAEVAESVKTIFKADNVPIEWEQVEVSGIADSAAPS
NCU00775.1	SDIFKPTKYGGKYTVTLIPG~DGIGAEVAESVKTVFKADNVPITWEQIEVSGVQDNASRT
FG05733.1 	SDIFKPAKYGGKYTVTLIPG~DGIGTEVAESVKTVFKADNVPVEWEQIEVSGLEG-AGRT
AN5790.1  	QDIFKPTKYGGKYTVTLIPG1DGIGAEVAESVKTIFKADNVPIEWEQVDVSGVDTGNKHS
          	.*****:************* ****:********:*******: ***::***:  .   :

MG01995.1 	GRTEDLFKESVASLRRNKLGLKGILHTPISRSGHQSFNVAMRQELDIYASISLIKNIPGY
NCU00775.1	---EEAFQQAVASLRRNKLGLKGILHTPISRSGHQSFNVAMRQELDIYASISLVKNLPGL
FG05733.1 	---EDAFRESVASLKRNKLGLKGILHTPISRSGHQSFNVAMRQELDIYASISLIKNIPGY
AN5790.1  	---EELFKESIASLRRNKLGLKGILFTPVERSGHQSFNVALRQELDIFASVVLIKNIPGY
          	   *: *::::***:**********.**:.**********:******:**: *:**:** 

MG01995.1 	ETRHKDVDLCIIRENTEGEYSGLEHQSVPGVVESLKIITRAKSERISKFAFSFALANNRK
NCU00775.1	QTRHKDVDLCIIRENTEGEYSGLEHQSVDGVVESLKIITRAKSERITKFAFQFALANQRK
FG05733.1 	ETRHKDVDLCIIRENTEGEYSGLEHQSVEGVVESLKIITRAKSERIARFAFSFALANGRS
AN5790.1  	KTRHDNVDLCIIRENTEGEYSGLEHQSVQGVVESLKIITRAKSERIAKFAFSFALANNRK
          	:***.:********************** *****************::***.***** *.

MG01995.1 	KVTCIHKANIMKLADGLFRSTFHQTAKEYPTLEANDMIVDNASMQCVSRPQQFDVLVMPN
NCU00775.1	KVTCIHKANIMKLADGLFRGTFNRLAKDFPQLECNDMIVDNASMQCVSKPQQFDVMVMPN
FG05733.1 	KVTCIHKANIMKLADGLFRSTFHQVAKDYPTLETNDMIVDNASMQAVSRPQQFDVMVMPN
AN5790.1  	KVTCIHKANIMKLADGLFRSTFHKTAENYPTLEVNDMIVDNASMQAVSRPQQFDVMVMPN
          	*******************.**:: *:::* ** ***********.**:******:****

MG01995.1 	LYGGILSNIGAALVGGPGIVPGCNMGRDVAVFEPGCRHVGLDIKGKDQANPTALILSGSM
NCU00775.1	LYGGILSNIAAALVGGPGVVPGCNMGRDVAVFEPGCRHVGLDIQGKDQANPTALLLSGTM
FG05733.1 	LYGGILSNIGAALVGGPGIVPGCNMGREVAVFEPGCRHVGLDIKGKDQANPTAMLLSGSM
AN5790.1  	LYGGILSNIGAALVGGPGVVPGCNMGRDVAVFEPGCRHVGLDIKGKDQANPSAMILSGSM
          	*********.********:********:***************:*******:*::***:*

MG01995.1 	LLRHLGLDDHANRISKAVYAVIAEG~KTRTRDMGGEATTNQFTKAILDKMETF-
NCU00775.1	LLRHLGLDDHANRISNAVYDVIAQG2KVRTRDMGGEASTHEFTRAILDSMEKSL
FG05733.1 	LLRHLGLDEHANRISKATYAVIADG2KVRTPDMGGKSTTHEFTRAILDKLETV-
AN5790.1  	LLRHLGLDDHANRISKAVYDVIGEG2KTRTRDMGGQATTHEFTRAVLDKMEAAL
          	********:******:*.* **.:* *.** ****:::*::**:*:**.:*
```
